# Land use change impacts on floods at the catchment scale: Challenges and opportunities for future research

**DOI:** 10.1002/2017WR020723

**Published:** 2017-07-02

**Authors:** M. Rogger, M. Agnoletti, A. Alaoui, J. C. Bathurst, G. Bodner, M. Borga, V. Chaplot, F. Gallart, G. Glatzel, J. Hall, J. Holden, L. Holko, R. Horn, A. Kiss, S. Kohnová, G. Leitinger, B. Lennartz, J. Parajka, R. Perdigão, S. Peth, L. Plavcová, J. N. Quinton, M. Robinson, J. L. Salinas, A. Santoro, J. Szolgay, S. Tron, J. J. H. van den Akker, A. Viglione, G. Blöschl

**Affiliations:** ^1^ Institute of Hydraulic Engineering and Water Resources Management Vienna University of Technology Vienna Austria; ^2^ Laboratory for Landscape and Cultural Heritage (CultLab), Department of Agricultural, Food and Forestry Systems (GESAAF) University of Florence Florence Italy; ^3^ University of Bern Bern Switzerland; ^4^ School of Civil Engineering and Geosciences Newcastle University Newcastle upon Tyne UK; ^5^ Division of Agronomy, Department of Crop Sciences University of Natural Resources and Life Sciences Tulln Austria; ^6^ Department of Land, Environment, Agriculture and Forestry University of Padova Padua Italy; ^7^ Laboratoire d'Océanographie et du Climat (LOCEAN, UMR 7159 CNRS/IRD/UPMC/MNHN) Paris France; ^8^ IDAEA, CSIC Barcelona Spain; ^9^ Professor Emeritus, Institute of Forest Ecology University of Natural Resources and Life Sciences Vienna Austria; ^10^ water@leeds, School of Geography University of Leeds Leeds UK; ^11^ Institute of Hydrology, Slovak Academy of Sciences Bratislava Slovakia; ^12^ Institute of Plant Nutrition and Soil Science, Christian Albrechts Universität zu Kiel Kiel Germany; ^13^ Department of Land and Water Resources Management, Faculty of Civil Engineering Slovak University of Technology in Bratislava Bratislava Slovakia; ^14^ Institute of Ecology, University of Innsbruck Innsbruck Austria; ^15^ Faculty of Agricultural and Environmental Sciences Rostock University Rostock Germany; ^16^ Department of Soil Science University of Kassel Kassel Germany; ^17^ Faculty of Science University of Hradec Králové Hradec Králové Czech Republic; ^18^ Lancaster Environment Centre, Lancaster University Lancaster UK; ^19^ Centre for Ecology and Hydrology Wallingford UK; ^20^ Computational Science Center, University of Vienna Vienna Austria; ^21^ Wageningen Environmental Research, Wageningen University and Research Wageningen Netherlands

**Keywords:** land use change, floods, catchment scale

## Abstract

Research gaps in understanding flood changes at the catchment scale caused by changes in forest management, agricultural practices, artificial drainage, and terracing are identified. Potential strategies in addressing these gaps are proposed, such as complex systems approaches to link processes across time scales, long‐term experiments on physical‐chemical‐biological process interactions, and a focus on connectivity and patterns across spatial scales. It is suggested that these strategies will stimulate new research that coherently addresses the issues across hydrology, soil and agricultural sciences, forest engineering, forest ecology, and geomorphology.

## Key Points

- Land use change impacts on floods are poorly understood at the catchment scale
- Numerous synergies are identified in exploring the effects of changed agricultural practices, artif. drainage, terracing, and forest cover
- A cross‐disciplinary systems approach aided by long‐term field studies and a focus on flow connectivity are needed to make major advances

## 1 Introduction

The frequency of major floods in many places around the world seems to be increasing [e.g., *Hall et al*., 2014; *Lins and Slack*, 1999]; flash floods occurred throughout Europe in June 2016; the Elbe and Danube flooded in June 2013, just 10 years after the 2002 “millennium” flood; in the UK there was severe winter flooding in both 2013/2014 and 2015/2016; and there are many more examples from all around the world such as the 2010/2011 Brisbane flood and the great flood in South Asia in 2016. Climate change may be a significant driver of changes in the flood frequency which has been widely investigated [e.g., *Merz et al*., 2014; *Hall et al*., 2014; *Viglione et al*., 2016]; however, as argued below, there are only a few studies on the role of land use change in modifying river floods.

Land use change has, potentially, a very strong effect on floods as humans have heavily modified natural landscapes. Large areas have been deforested or drained, thus either increasing or decreasing antecedent soil moisture and triggering erosion. Hillslopes were modified for agricultural production, thus changing flow paths, flow velocities, and water storage, and consequently flow connectivity and concentration times. The intensification of agricultural practices has resulted in the formation of platy dense soil horizons with preferential lateral flow which may reduce and/or retard vertical infiltration in the soils, but cause an intensification of lateral mass flow besides the reduced filter and buffer processes in deeper soil horizons. It is likely that hydrologically significant changes will continue in the next decades due to loss of agricultural land and forests [*Wheater and Evans*, 2009]. In all of these processes, however, the exact role of land use change in modifying river floods is still elusive.

Studies that examine the impact of land use changes on streamflow and floods often obtain contradictory results for the same kind of change. Although results from individual studies are legitimate, it is difficult to obtain general statements on the impacts since each study takes a rather narrow and study specific perspective. Some recent publications such as the paper of *Gupta et al*. [2015] on the relative impacts of climate and land use changes on streamflow or that by *Alila et al*. [2009] about the effects of forest practices on floods have triggered scientific debates with the results being criticized by many scientists. Such debates clearly show the need for new approaches in this field and the need to gain more quantitative insights into land use change effects on flood generation at the catchment scale.

In this commentary, the main research gaps concerning the impact of land use change on floods are identified, and strategies for addressing them are proposed. The focus of this paper is on the role of agricultural practices, drainage, terracing, and forest change. The impacts of land use change due to urbanization are also important, but are not further considered in this paper, because processes related to urbanization [e.g., *Hollis*, 1975] are better understood and easier to measure compared to the other land use change types and because effects of urbanization are generally of more local nature. Issues addressed in this commentary are how flood generation is modified, at what scale and in what hydrological context, with particular attention to process feedbacks, drawing from ideas in hydrology, soil and agricultural sciences, forest sciences, and geomorphology. It is hoped that the research directions proposed here will stimulate new research that addresses the issues in a coherent way across these disciplines.

## 2 A Tangled Web of Interactions

### 2.1 Process Interactions Across Time Scales

**Figure 1:**
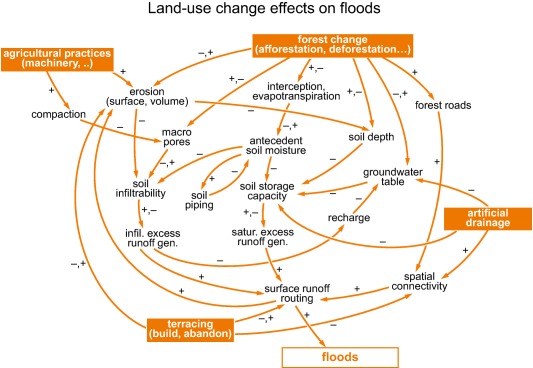
Schematic of process interactions in land use change effects on floods at the catchment scale. Plus and minus signs indicate whether an increase in a variable increases or decreases another variable. The processes shown here are imbedded in a broader context of environmental and socioeconomic processes.

Floods are the outcome of coupled processes with widely diverging time scales [*Gaal et al*., 2012] that are all, to some degree, influenced by land use. Land use change impacts on floods therefore involve a plethora of closely intertwined process dynamics that make their analysis and the prediction of any impacts at the catchment scale extremely challenging. The schematic in Figure 1 presents a conceptual view of some of the process interactions considered to be relevant. For example, clear‐cutting in forest plantations decreases interception and evapotranspiration which increases antecedent soil moisture and consequently decreases soil storage capacity [*Brown et al*., 2005]. The use of heavy machinery on agricultural land tends to cause soil compaction and a decrease in soil infiltration, resulting in increased surface runoff. The process interactions involve a number of positive feedbacks enhancing small disturbances and negative feedbacks, where the effects of disturbances are dampened due to counteracting processes. An example of a positive feedback is erosion caused by agricultural intensification resulting in a reduction of soil depth, a reduction of soil storage capacity, and an enhancement of surface runoff which in turn increases erosion [*Zink et al*., 2011]. An example of a negative feedback and how it might change with time is related to the interaction of deforestation with soils: initially there may be an increase in soil moisture but, for internally erodible soils, this may result in the development of subsurface pipe systems, which in turn may reduce soil moisture and therefore reduce flood generation [*Wilson et al*., 2013].

The time scales involved in the process interactions may range from event to seasonal to centennial scales. For example, drainage of peatlands may initially result in lower water tables and an increase in water storage capacity. However, oxidation of peat triggered by lower water tables results in loss of peat thickness over time, which then reduces the storage capacity. The short‐term response to drainage may therefore be different from the long‐term response which is more strongly affected by feedbacks [*Acreman and Holden*, 2013]. When agricultural practices change, the topsoil characteristics may respond very quickly, while the subsoil may respond more slowly, and both are modulated by seasonal fluctuations of biotic activities associated with the energy and water balances. The coupling across different time scales adds complexity to the catchment system making cause‐effect relationships less obvious. Critical transitions or tipping points may occur, leading to sudden changes in the system behavior [*Nicolis and Nicolis*, 2007; *Sivapalan and Blöschl*, 2015]. These changes are even more likely to occur if socioeconomic processes play a role, which requires broadening the perspective beyond that of Figure 1. For example, if widespread soil erosion occurs on arable fields, farmers may decide, for economic reasons, to convert the fields to permanent pasture, which in turn affects the runoff response of the landscape [*Cerdà et al*., 2009]. Other processes not included in Figure 1 are those associated with land‐atmosphere feedbacks, such as the coupling of land use changes with the local climate.

### 2.2 Upscaling, Spatial Connectivity, and Scale Dependency

Spatial scales are equally important as time scales when attempting to understand land use change impacts on floods. Below we are referring to three different spatial scales following *Dooge* [1982, 1986]: the local or plot scale (1 m), the hillslope scale (100 m), and the catchment scale (10 km or larger). Most field research has been performed at the plot scale and upscaling observed effects from plots to hillslopes and catchments has proven to be difficult [*O'Connell et al*., 2007].

While, in the past, the emphasis of upscaling has usually been on understanding random spatial variability, the spatial connectivity of flow processes is now increasingly recognized as a key determinant of land use change effects [*Blöschl et al*., 1995; *Western et al*., 1998; *Van Dijk et al*., 2005; *Fraser et al*., 2013; *Band et al*., 2014; *Pfister et al*., 2015]. Forest roads, for example, may increase floods by creating preferential paths of overland flow [*Gucinski et al*., 2001; *Guzman et al*., 2017]. On agricultural fields, the trafficked crop interrows usually experience a much stronger seasonal compaction than the crop rows, resulting in disconnected patterns of soil infiltrability which may enhance surface runoff [*Silgram et al*., 2010]. Understanding the influence of the spatial organization of patches and the interaction with linear structures such as field borders, ditches, and ephemeral gullies on the overall catchment response is particularly challenging, yet essential for upscaling the effects of soil compaction to the catchment scale. Owing to the multiple time scales involved, the coevolution of vegetation and soils may lead to emergent spatial features such as subsurface flow networks [*Band et al*., 2014]. Connectivity is also relevant at the subcatchment scale. If, for example, a tributary catchment is drained and the effect within that tributary is to reduce the flood peak and increase the lag times between a precipitation peak and the streamflow peak, then this conversely can cause an increase in the main channel flood peak if the timing of the two peaks (tributary and mainstream) become synchronous [*Holden*, 2005].

Owing to the spatial variability of flow processes, land use change effects on floods vary with the catchment scale. The impact of land use changes usually decreases with increasing catchment area for a finite size of the disturbance [*Blöschl et al*., 2007] although the exact relationship depends on the local setting [*Bathurst et al*., 2011]. Both the flow aggregation behavior at the hillslope scale and the relative importance of hillslope routing versus channel routing control the scaling of impacts with catchment area.

As the catchment scale increases, it becomes more difficult to identify any land use change effects on floods from observed discharge data due to multiple controlling factors and process interactions [*Viglione et al*., 2016]. The effects of agricultural practices on the topsoil structure are very clear at the plot scale but less clear at the catchment scale [*Hess et al*., 2010] and, similarly, the effects of drainage practices may be less evident as the catchment scale increases [*Robinson*, 1990; *King et al*., 2014]. Paired catchment studies usually show a stronger impact of land use changes on the seasonal water balance than on floods [*Brown et al*., 2005]. One reason is the masking of land use change effects by other processes as the catchment scale increases, such as the variability in precipitation patterns [*O'Connell et al*., 2007]. Other processes, such as human settlement dynamics, may additionally confound causal relationships between land use changes and floods [*Bradshaw et al*., 2007].

Against the backdrop of the issues of scale and scaling, below the most important research gaps for each of the land use change types are discussed.

## 3 Research Gaps

### 3.1 Forest Changes

Since the Neolithic age, forests have been lost to cropland and grassland at large scales [*Gaillard et al*., 2015]. In the twentieth century, forest cover has changed worldwide with different trends. In some areas, such as the tropics, forest cover has decreased as a result of logging and expansion of agriculture and urban infrastructure, while in other areas, such as in Mediterranean landscapes, forest cover has typically increased as a consequence of abandoning agricultural lands in hilly and mountainous areas for economic reasons, allowing for natural regrowth of forest. Globally, 2.3 × 10^6^ km^2^ of forest was lost in the years 2000–2012 and 0.8 × 10^6^ km^2^ of new forest was gained [*Hansen et al*., 2013]. Furthermore, forest management practices have changed almost universally due to the introduction of new machinery and due to a change in perception from a pure exploitation of forests toward a preservation of their function due to an increase in ecosystems awareness [*Messier et al*., 2015; *Teuffel et al*., 2005].

The effects of forest cover on the flood regime are ambiguous. Experimental studies show that forest cover, compared to grassland, may reduce average catchment discharge as a result of (i) increased rainfall interception, (ii) increased transpiration, (iii) reduced soil moisture, and (vi) increased permeability of soils [e.g., *Brown et al*., 2005; *Andréassian*, 2004]. The effects of forest cover on flood peaks are more difficult to isolate. Plot‐scale studies suggest that forest cover may lead to lower and more delayed flood peaks compared to cropland and grassland as a result of the aforementioned processes. These effects tend to be limited to small and moderate rain storm events [e.g., *Brown et al*., 2005; *Bathurst et al*., 2011] although, in some settings, forests may reduce flood frequency over the full range of event magnitudes [*Alila et al*., 2009]. At the catchment scale, the impact of forest change on flood peaks is less well understood compared to the plot and hillslope scales due to a scarcity of experimental data. The main difficulties lie in the nonlinearity of runoff generation processes and in the nonstationarities introduced by forest changes. These issues need to be better understood both across scales and between scales.

Effects of forest change that are more indirect include increased surface runoff on forest roads and increased soil erosion and the development of gullies after deforestation which both may enhance floods in steep terrain [e.g., *Vose et al*., 2011] and increased snow accumulation and hence snowmelt in deforested regions [*Bernsteinová et al*., 2015]. Vegetation‐related controls on flood generation mechanisms at the hillslope scale are less well understood than those at the plot scale. Soil hydraulic conductivity, macropores, and cracks are often thought to be more relevant for the infiltration excess mechanism, and soil depth may be more important for saturation excess, but the universality of this finding is not clear [*Rawlins et al*., 1997]. Root water uptake of woody plants is usually from deeper soil water sources than that of herbaceous plants, so moisture depletion is not necessarily from the surface where it strongly matters for runoff generation. In addition, it has been recently shown that plants and streams often do not use the same water pools [*Evaristo et al*., 2015].

While it is clear that the soil structure responds slowly to deforestation, afforestation, and reforestation, the exact time scales related to such changes and their controls are not well understood. It is necessary to understand better how fast preferential flow pathways in the subsurface evolve, but there is currently a lack of simple methods for quantifying these flow pathways in the field. Another open question is how forest management activities, affecting the age and composition of the forest, translate into changes in soil structure and consequently soil moisture. Clearly, soil moisture affects flow paths, including the type of runoff generation mechanism (overland flow versus subsurface stormflow), but more research in experimental catchments is needed to better understand the impacts on flood generation and on the frequency and magnitude of floods. Finally, forest fires and waxy leaf litter may lead to hydrophobic soil surfaces [*Vieira et al*., 2015], and impermeable crusts may develop under the direct impact of raindrops on bare soils after forest removal. The influence of both processes on flood generation is an important research gap.

### 3.2 Soil Compaction due to Agricultural Practices

Globally, about 680,000 km^2^ of agricultural land is affected by soil deformation (including compaction and shearing), mostly because of poor agricultural practices such as using heavy machinery [*Batey*, 2009]. Plot‐scale soil deformation has been extensively studied in the context of its negative effects on agricultural yield, soil quality, and soil permeability [*Dörner and Horn*, 2006; *Zink et al*., 2011; *Alaoui et al*., 2011] as well as in terms of transport of contaminants in the vadose zone [*Iversen et al*., 2012; *Holman et al*., 2003]. A large number of studies have also investigated the impacts on runoff generation at plot and hillslope scales [*Deasy et al*., 2014]. However, the effect of soil deformation on floods at the catchment scale has received much less attention. Typically, the effect of agricultural practices on the flood regime has been determined by projecting known plot‐scale impacts, e.g., modification of soil surface coverage and soil hydraulic properties by tillage and subsequent changes in infiltration capacity, onto catchment hydrological effects by conjecture [*Schwen et al*., 2011; *Gieska et al*., 2003; *Fraser et al*., 2013; *Zumr et al*., 2015] or statistical approaches [*Potter*, 1991]. Most hydrological models assume soil hydraulic characteristics not to change with time, although they usually do change due to tillage in the short term and in the intermediate term due to accumulation of subsoil compaction causing reduction of infiltration capacity and rooting depth. There is a need to better understand the dynamic nature of soil structure and its effects on hydrology. In particular, the questions how the seasonal variations of soil hydraulic properties are modified by tillage, compaction, cracking by repeated shrinking and swelling (on fallow fields or bare fields in winter), and soil sealing processes and how this affects runoff at scales larger than the plot scale require further investigation. A mechanistic description of coupled mechanical and hydraulic processes is needed that captures the evolution of soil structure by tensile forces (crack generation and propagation) and compressive and shearing stresses (wheeling), particularly in tilled horizons, but also down to deeper depth, and the change of hydraulic functions with deformation and state of the soil structure. The description should also include biological effects on the soil structure characteristics such as preferential flow pathways through macropores induced by earthworms and root penetration, and hydrophobicity induced by fire or fungi [*Band et al*., 2014]. Accounting for exchange fluxes between macropores and soil matrix [*Alaoui and Goetz*, 2008] and extending the common, but flawed, assumption of static hydraulic properties in Richards equation‐based models toward seasonally dynamic hydraulic properties, would improve plot‐scale models of water flow in soils and therefore estimates of surface runoff. How to incorporate these effects in catchment‐scale models in a meaningful way is, however, another unresolved issue.

The response of soils to varying agricultural practices is a multiscale process. While soil deformation leads to immediate changes of soil properties [e.g., *Leitinger et al*., 2010; *Hartge and Horn*, 2016], soil regeneration may occur with lags of several years, decades, or even centuries [*Peng and Horn*, 2008]. Even when agricultural fields are abandoned, compaction effects in the subsoil may still be measureable after decades or longer [*Kellner and Hubbart*, 2016], indicating a long‐memory effect. Potential factors controlling this memory effect are land use, soil types, topography, and climate which all need to be elucidated at the appropriate time scales.

### 3.3 Artificial Drainage

Over the past century, around 2,000,000 km^2^ of land have been drained globally [*Framji et al*., 1982] to lower the groundwater table in order to convert wetlands into farmland and to enhance growing conditions for forests [*Fohrer et al*., 2007]. A large amount of research has been published concerning how drainage affects the water balance, water quality, and salinity [e.g., *Tiemeyer et al*., 2006; *Duncan et al*., 2008], but there has been only little research on the impacts of drainage on flood generation [*Changnon et al*., 1996]. The effect on floods depends on numerous local factors. In low permeability soils (with high clay contents) where groundwater tables are high, artificial drainage may lower the water table, thus increasing storage capacity which may reduce floods. In high permeability soils, faster transmission of the subsurface water flow in the drainage system may increase peak discharges [e.g., *Zucker and Brown*, 1998]. In wetland‐dominated areas, artificial drainage may connect formerly isolated marshes and increase flood flows [*Blann et al*., 2009]. Whether artificial drainage increases or decreases flood peaks also depends on the drain types—open ditches or pipes [*Rycroft and Robinson*, 2008]—and the event magnitudes [e.g., *Acreman and Holden*, 2013; *Rahman et al*., 2014]. An additional problem in determining the impact of specific subsurface drainage systems on floods is that they have often been built over periods of centuries and, therefore, their location and efficiency are unknown. Potential locations of drainage pipes have been identified by soil and topographic information as proxies but, in the absence of local measurements, it is difficult to estimate their efficiency. It would be crucial to obtain generic relationships of the decay of pipe efficiency as a function of soil, climate, and land use controls. Similar to the effect of soil compaction, the effect of artificial drainage on runoff generation may be time delayed [e.g., *Holden et al*., 2006]. Immediate effects of management interventions are not necessarily those that occur in the longer term. Representative monitoring studies should therefore be carried out over decades rather than years. Most monitoring is conducted for case studies and it is difficult to generalize beyond the site‐specific conditions. In order to generalize and upscale observed effects, it would be necessary to better understand the individual controls on flood response to drainage as a function of soil hydraulic characteristics, preferential flow paths, location and dynamics of the groundwater table, recharge and the drainage properties.

### 3.4 Terracing

Although no reliable global inventory is readily available, the construction of terraces has been widespread throughout the world for millennia to facilitate cultivation, harvesting, and irrigation, reduce soil erosion, and increase soil storage capacity [*Dotterweich*, 2013; *Gallart et al*., 1994; *Dagnew et al*., 2015]. Owing to the flatter topographic slope, surface runoff is typically delayed by the presence of terraces thus reducing peak flows, but terraces may also enhance saturation excess runoff thus increasing flood peaks [*Gallart et al*., 1994]. Terraces may also affect floods more indirectly through reducing shallow landslides [*Agnoletti et al*., 2012]. Since flooding is rarely the main concern when terraces are constructed, knowledge of their effects on runoff generation and routing is rather limited. Important research questions therefore involve the effect of the different types of terraces and their state on the hydrological processes at the hillslope scale for different climatic and soil conditions. This research should also include the impacts of different drainage practices that were used in terracing and the impacts of human modifications of terrace walls and surfaces (e.g., stone mulching) on terrace stability and on flood generation. More recently, terraces have been widely abandoned as maintenance and cultivation are becoming less economically viable [*Agnoletti*, 2013] and, often, historical terraces are intentionally dismantled when ditch irrigation is replaced by drip irrigation in a completely new geomorphologic and pedogenetic setting [*Dudal*, 2005]. Abandonment of terraces may lead to geomorphologic changes on the hillslopes and in the drainage system. Gullies may develop and the old natural water pathways across the terraces may be reactivated, sometimes accompanied by piping phenomena [*Romero‐Díaz et al*., 2016]. Such changes may be episodic after extreme erosive events or more gradual. The hydraulic and pedologic properties of flow concentration, changes in erosion, local redistribution of topsoil and formation of new soil types should be assessed and monitored in order to get a better insight into these processes. In fact, this could be seen as a unique opportunity to witness an experiment of nature and learn more about soil and flow network formation.

## 4 Possible Ways Forward

Understanding the impacts of land use change on flood generation across different space and time scales requires a new research thrust. Even though the research questions in the land use change categories discussed here are quite diverse, common threads emerge. Systems thinking to link processes across time scales, controlled long‐term field experiments at the plot scale, a focus on connectivity and spatial patterns, and organizing a coherent research theme within and across disciplines are believed to be the pillars of progress in this area.

### 4.1 Complex Systems Thinking to Link Processes Across Time Scales

The seemingly overwhelming complexity of land use change effects as evidenced by the diverse and often contradictory research results published in the literature may perhaps be best addressed by adopting a broader perspective of system approaches that explicitly quantify the interactions of processes across multiple spatiotemporal scales, drawing methodological inspiration from nonlinear geophysics [*Pires and Perdigão*, 2015] and specific process understanding from the various disciplines. A systems view of these processes can be developed by integrating the process understanding within hydrology, soil science, agricultural engineering, forest ecology, and geomorphology, in order to obtain a common framework, as is already done in Earth System Modeling. Analysis steps may include the following: (i) Merging the process understanding from the disciplines, e.g., by starting from causal loop diagrams (similar to that in Figure 1) of already detected and hypothesized interactions for specific land use change settings, and identifying the most important state variables that are necessary to describe the dynamical system. A key focus needs to be on the memory effects of the system components that influence process interactions, e.g., through time‐delayed changes in the soil structure. (ii) Grouping land use changes by their types, such as terrace types and by their hydrological setting, and ranking of important feedback mechanisms in different settings. (iii) Plot‐scale dynamic models based on the causal loops would be a starting point for quantifying the process interactions at fast and slow time scales. Since parameter estimation and testing of system models are inherently difficult, specific strategies are needed. These may include a focus on better understanding model complexity, the use of long‐term multivariable data sets, and comparative approaches that exploit spatial gradients to infer the dynamic behavior of the catchment system with respect to land use change (i.e., trading space for time in complex systems with nonlinear process coevolution [*Perdigão and Blöschl*, 2014]). Starting from a complex systems view, research should also include modeling frameworks of the “spherical cow” [*Harte*, 1988] type, which identify simplified treatments of complex problems to bring out their main characteristics.

### 4.2 Long‐Term Experiments on Physical‐Chemical‐Biological Process Interactions

There is a long history of plot‐scale experiments in soil and agricultural sciences, paired catchment studies in forest science, and short‐term field experiments and long‐term catchment monitoring in hydrology and geomorphology. These different strands of research need to be better integrated in order to test hypotheses on land use change effects on floods, assisted by system models. A number of factors will be central to the integration: (i) Long‐term observations of variables that are directly relevant for understanding land use change effects are needed, such as the joint mechanical and hydraulic properties of soils as a function of agricultural activities, or changes in the efficiency of drainage pipes. Tailored field experiments, such as lysimeters and measurements of surface runoff on sloping (and convergent) ground would be part of the monitoring setup. (ii) Catchment‐scale studies (including paired catchments) should not only address afforestation/deforestation but also other land use changes (agricultural practices, drainage, and terracing). (iii) Similar to system models, a focus on memory effects (e.g., in soil structure after long‐term agricultural use) is needed, facilitated by long‐term observations of, for example, change in soil structure and decay of terraces. (iv) Confounding factors may mask the land use change effects. If possible, controlled and/or known boundary conditions should therefore be established in order to enhance the comparability and repeatability of individual studies. Globally, there is a strong effort being made in a number of environmental disciplines toward multivariable long‐term observations, such as in ecology and critical zone research, and a similar trend can be observed in hydrology [*Holländer et al*., 2009; *Zacharias et al*., 2011]. Since individual research groups often lack the capacities for maintaining such observatories, it may be strategic to establish observatories that act as collaborative platforms for a number of research groups. In a similar vein, it may be useful to maintain, repurpose, and extend existing long‐term field sites designed for other science questions such as nutrient transport, erosion, soil compaction, and agricultural yield. In some instances, historical information (e.g., from geology, geoarchaeology, and historical documentary) may be very useful, e.g., to explore documentary evidence of increased soil erosion after the abandonment of terraces and its relationship to historical floods.

### 4.3 A Focus on Connectivity and Patterns Across Spatial Scales

Spatial‐scale issues from the plot to the catchment scale have been the main barrier to progress in identifying causal relationships between land use change and floods. It is believed that major progress can come through adopting connectivity of flow paths and their spatial patterns as unifying themes in identifying causal mechanisms. While the local inputs will differ, depending on the type of land use change and hydrological context, similarities in the aggregation behavior along the flow paths may suggest similarities in the effects of soil compaction, drainage, terracing, and forest change. Connectivity should be treated as a dynamic rather than a static characteristic of catchments, implying that not only flow connectivity at the flood event scale is of interest but also how it evolves over time, e.g., due to soil physical processes, erosion and decaying drainage systems. Five steps are envisaged in the analysis: (i) Characterization of flow connectivity at different spatial scales with a focus on the dynamic response of soils and land cover to extreme precipitation events, patterns of infiltration capacity and surface runoff. High‐resolution remote sensing methods such as Lidar or ground penetrating radar, and terrestrial methods such as distributed temperature sensing, may assist in characterizing the connectivity structure and the flow paths. (ii) Developing macroscale conceptualizations of flow resistance (both on the surface and in the subsurface) that represent the effective behavior of small‐scale land use related features (e.g., terraces and drainage systems) not explicitly resolved at the scale of catchment models. (iii) Quantifying the effects of land use changes on connectivity, and identifying the factors controlling the importance of the location of the disturbance relative to the topography and the catchment outlet, based on modeling and field studies. This may lead to inferential relationships of how land use changes modify the spatial organization of the flow paths. (iv) Analyzing the effect of changes in connectivity on changes in floods, again based on modeling and field studies. (v) Deriving scaling relationships or parameterizations to upscale local‐scale land use change impacts to the catchment scale, as a function of flow connectivity and flow dimensionality (1‐D, 2‐D, or complex 3‐D flow patterns).

### 4.4 Generalization: Toward a Coherent Research Thrust

As, ultimately, one would like to understand the generic principles underlying flood response to land use changes rather than those of one particular catchment, it is essential to generalize the findings obtained locally. Generalization can move along a number of complementary avenues. (i) A typology could be developed to assist in the synthesis in terms of types of land use changes (e.g., terrace types), runoff generation mechanisms, and other hydrological characteristics such as soils, vegetation, topography, landscape type, and climate. A common framework for organizing the results, including criteria of similarity, will need to be implemented for such an analysis. (ii) For all land use change categories, a meta‐analysis of reported studies would be useful to synthesize the findings from the existing literature. This analysis could also be used to rank the studies by the magnitudes of their impact which would assist in identifying worst‐case scenarios of land use change impacts on flood generation, and it could form the basis for a data‐based regionalization of land use change impacts on floods as a function of scale. There are some attempts of meta‐analyses in hydrology [e.g., *Blöschl et al*., 2013; *Mutema et al*., 2015], but the specific settings of hydrological studies often make it difficult to draw general conclusions, in particular if not enough information is reported in the related publications. A more complete and consistent reporting of relevant information is therefore needed as is already standard in other disciplines [*Koutsoyiannis et al*., 2016]. (iii) Collaborations between hydrology, soil science, agricultural science, forest science, and geomorphology already exist in a number of research contexts. For example, soil scientists have already invested considerable efforts in developing pedotransfer functions for upscaling soil parameters to the catchment scale, and hydrologists have embraced forest ecological concepts as part of eco‐hydrological research. However, more coherent and enduring collaboration through multidisciplinary research consortia is needed with shared hypothesis building, experimentation, and data analysis. Using the same scientific terminology, simplified approaches, data accessible to the entire group, and joint conceptualizations of core processes would all help strengthen the ties. This trend will likely be assisted by open data policies that are becoming more common [*Montanari et al*., 2013].

## 5 Conclusions

As humankind is witnessing increasing floods in many places around the world there is an urgent need to better understand one of the critical drivers of flood regime changes. Numerous synergies have been identified here in addressing research gaps of understanding the effects of changes in agricultural practices, drainage, terracing, and forest management on floods. Clearly, there is a need to fully exploit the experience gained in the past in diverse fields such as hydrology, soil and agricultural sciences, forest science, and geomorphology, and come up with a coherent research thrust. The most promising progress is expected through four avenues: (i) Complex system approaches to the coevolution of the landscape structure at varying time scales [*Perdigão and Blöschl*, 2014] will be the basis of a more holistic approach of deciphering the many process interactions relevant for flood generation, and in particular the role of catchment memory. (ii) Long‐term experiments at the plot, field, and catchment scales that explore processes important for land use change impacts on runoff generation are essential, preferably with controlled or at least known boundary conditions. (iii) Major progress can come through adopting connectivity of flow paths and spatial patterns as unifying themes in identifying causal mechanisms and assisting in upscaling the mechanisms from the plot to catchment scales. (iv) Finally, coherence among the research community both within the disciplines and across disciplines should be fostered by better collaboration, e.g., through promoting meta‐analyses, and through research consortia in order to make progress in this important area of environmental research. A better connected community is likely to make major headway in understanding the role of land use change in recent floods, which would be a robust starting point for predicting changing floods in the future.
